# Small Bowel Adenocarcinoma: From Molecular Insights to Clinical Management

**DOI:** 10.3390/curroncol29020104

**Published:** 2022-02-17

**Authors:** Fabio Gelsomino, Rita Balsano, Stefania De Lorenzo, Ingrid Garajová

**Affiliations:** 1 Department of Oncology and Hematology, Division of Oncology, University Hospital of Modena, 41124 Modena, Italy; 2 Medical Oncology Unit, University Hospital of Parma, 43126 Parma, Italy; rita.balsano@gmail.com (R.B.); ingegarajova@gmail.com (I.G.); 3 Oncology Unit, Azienda USL Bologna, 40124 Bologna, Italy; stefania.delorenzo@libero.it

**Keywords:** small bowel, molecular alterations, genomic profiling

## Abstract

Small bowel adenocarcinoma (SBA) is a rare malignancy, with a rising incidence in recent decades, and accounts for roughly 40% of all cancers of the small bowel. The majority of SBAs arise in the duodenum and are associated with a dismal prognosis. Surgery remains the mainstay of treatment for localized disease, while systemic treatments parallel those used in colorectal cancer (CRC), both in the adjuvant and palliative setting. In fact, owing to the lack of prospective data supporting its optimal management, SBA has historically been treated in the same way as CRC. However, recent genetic and molecular data suggest a distinct profile from other gastrointestinal malignancies and support a more nuanced approach to its management. Herein, we briefly review the state-of-the-art in the clinical management of early-stage and advanced disease and recent discoveries of potentially actionable genetic alterations or pathways along with the most promising ongoing clinical trials, which will hopefully revolutionize the treatment landscape of this orphan disease in the foreseeable future.

## 1. Introduction

Despite the small bowel representing about 75% of the length of the intestinal tract and over 90% of the surface area of the alimentary tract, small bowel adenocarcinoma (SBA) is a very rare tumor. Since diagnosis of SBA is often difficult due to aspecific symptoms, the discovery of disease at a late stage is very frequent. The prognosis remains dismal, with a life expectancy at 5 years of 14–33% [1].

## 2. Epidemiology

SBA accounts for less than 5% of gastrointestinal cancers [2]. Historically, it has been the dominant small intestinal histology, followed by neuroendocrine tumors, lymphoma, and sarcoma (most commonly gastrointestinal stromal tumors and leiomyosarcoma). In the following years, neuroendocrine tumors became the most frequent histology, especially in distal segments of the small intestine. The overall incidence of SBA is slowly increasing, with a mean age at diagnosis of 60 years and a prevalence in men [3]. The most involved segment is the duodenum (60%), followed by the jejunum (25–29%) and ileum (10–13%) [1,3,4]. The factors responsible for this different localization are not completely understood, but it has been supposed that the exposure to bile and its metabolites, due to the presence of the ampulla of Vater, might at least in part explain the differences [5]. 

## 3. Etiology

A number of risk factors and predisposing conditions has been described for SBA. Among lifestyle factors, alcohol consumption, smoking, and obesity are associated with an increased risk of this tumor [6]. Diet plays a relevant role, as a higher incidence of SBA has been found in consumers of carbohydrates, red meats, and lower intake of coffee, fruit, and vegetables [7]. Dietary risk factors are similar between SBA and CRC; nevertheless, the lower incidence SBA is probably caused by the short transit time in the small intestine with less contact with carcinogens. Chronic inflammation is correlated with the SBA etiology. Inflammatory bowel diseases, in particular Crohn disease, increase the incidence of SBA within the involved area of the small intestine. The risk augments with both the extent of the small bowel involvement and the duration of disease. Palascak-Juif et al. reported a cumulative risk of SBA of 2.2% after 25 years of Chron’s disease [8]. Patients who underwent small bowel resection have a lower risk of SBA [9]. Furthermore, patients affected by coeliac disease have a risk of developing an SBA of about 8% [10]. Other conditions associated with an increased risk of SBA are cystic fibrosis, as well as different hereditary cancer syndromes, such as hereditary non-polyposis colorectal cancer (HNPCC), Peutz-Jeghers, and familial adenomatous polyposis (FAP) [11,12,13,14].

## 4. Molecular Alterations

**Figure 1:**
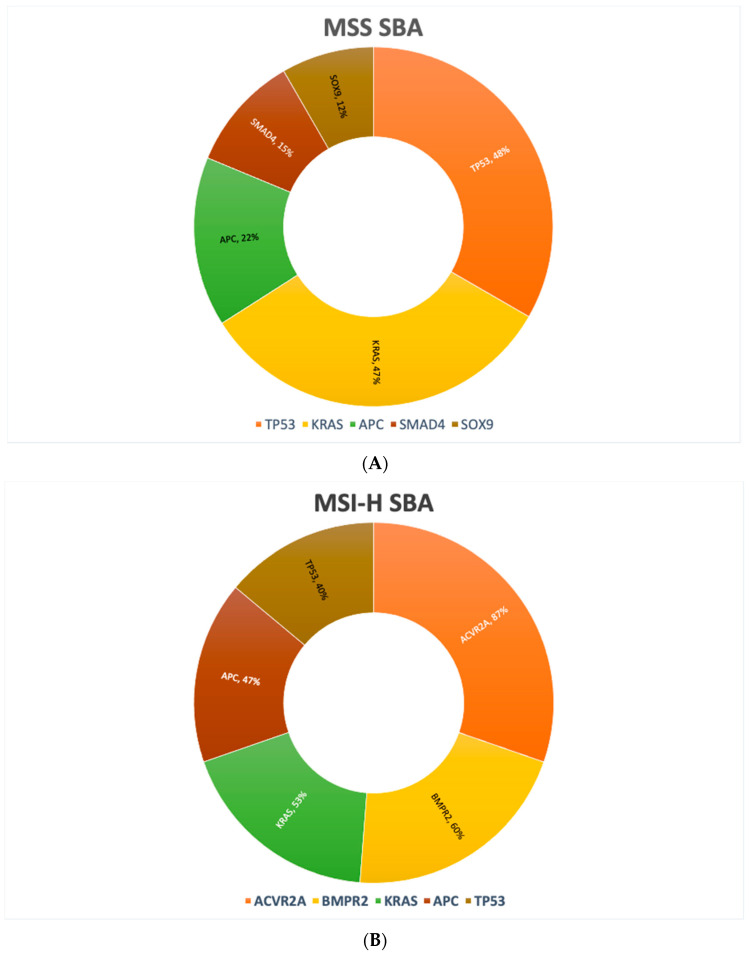
(**A**) Most frequently altered genes in MSS SBA. (**B**) Most frequently altered genes in MSI-H SBA. Abbreviations: MSS, microsatellite-stable. MSI-H, microsatellite instability-high.

Molecular profiling has gained momentum in the characterization of different cancer types. Various gene alterations might be druggable and can drive therapeutic decision in patients with several metastatic cancers. Recent studies have made major strides in understanding the molecular drivers of SBA. Schrock et al. reported results of their large cohort study in which genomic profiling was performed in 7559 cancer patients, including SBA, gastric cancer (GC), and CRC. Genomic profiles were analyzed on either a 236 or 315 cancer-related gene panel, and alterations were compared across these three tumor types. Interestingly, SBA has a different genomic profile compared to GC or CRC, with a different incidence of various genetic alterations [15,16]. These variations might contribute to the disparities in patients’ outcomes and treatment resistance. The most evident differences between SBA and CRC included frequency of genomic alterations in the APC and CDKN2A gene. GC profiles differed from those of SBA, mostly in frequency of genomic alterations in KRAS, APC, and SMAD4 genes [16]. In a separate report, exome sequencing performed on 106 primary SBAs were analyzed in detail [15]. The authors reported the most frequently mutated genes, both in microsatellite stable (MSS) (85.8%) and microsatellite instability-high (MSI-H) SBAs (14.2%), as shown in Figure 1A,B. Table 1 summarizes the most frequent gene alterations of SBA, GC, and CRC.

Of note, no statistical differences were observed among the top five most commonly altered genes (TP53, KRAS, APC, SMAD4, PI3KCA) when comparing duodenal adenocarcinoma with cancers of other small bowel sites, thus supporting the hypothesis that anatomic differences are the main driver of the worst patient outcome when comparing duodenal versus jejunal/ileal adenocarcinomas [16]. Furthermore, in another study, chromosomal copy number aberrations were compared between SBA, CRC, and GC. Hierarchical clustering revealed substantial overlap of 27 SBA copy number profiles with matched CRCs, but less overlap with profiles of GC, suggesting that MSS SBAs are more similar to CRC than to GC [17]. Moreover, the genomic profile of SBA diverges from that of CRC, also in the type of mutations. For example, BRAF mutations characterize about 10% of CRCs, with a similar rate in SBAs, however, while in CRC, BRAF V600E is by far the predominant mutation, it affects only 10.3% of BRAF-mutant SBAs [17,18]. Additionally, a slightly higher rate of alterations of HER-2 have been observed in SBA as compared to CRC (9.5% vs. 5.1%); however, while mutations prevail in SBA (70–76%), the majority of alterations in CRC (66%) are amplifications [3,17]. Microsatellite instability (MSI) is a marker of a deficient mismatch repair (MMR) system that represents a molecular hallmark of HNPCC, also known as Lynch syndrome, which is usually linked to a germ-line mutation in one of the MMR genes [19]. However, while the majority of cases of MSI-H CRC is sporadic, usually linked to an epigenetic inactivation of hMLH1, the MSI-H phenotype seems more common in SBA (12–35%), but the proportion of Lynch syndrome among MSI-H tumors is higher, especially in earlier-stage disease [20]. Of note, studies of celiac-associated SBA, although limited by the small number of patients enrolled, have consistently demonstrated a higher rate of MSI-H tumors, ranging from 50% to 73% [3]. 

## 5. Clinical Presentation and Initial Diagnostic Workup

Diagnosis of SBA is often insidious, because of the aspecific presentation. The most common symptoms are abdominal pain, weight loss, dyspepsia, diarrhea, nausea, vomiting, bloating, fatigue, and gastrointestinal bleeding. Sometimes, iron deficiency anemia from occult gastrointestinal bleeding may characterize the onset of the disease. SBA can lead to specific complications, depending on the tumor location, including jaundice, obstruction, and perforation. These symptoms can mimic other more common benign conditions, such as cholelithiasis, cholangitis, pancreatitis, inflammatory bowel disease, or appendicitis, which can explain a mean symptom-to-diagnosis interval that can approach 2 years [21]. Furthermore, the limited sensitivity of conventional radiological imaging may significantly contribute to the delayed diagnosis [22,23]. Moreover, while a significant proportion of CRCs is screen-detected, the vast majority of SBAs presents with a local complication in an emergency situation, most often gastric outlet or biliary obstruction for duodenal tumors or cramping abdominal pain and intestinal obstruction for jejuno–ileal SBAs, which require specific management (i.e., palliative surgical diversion or endoscopic biliary stenting in patients with non-resectable disease, depending on tumor location). Therefore, it is not surprising that SBA presents with a more advanced disease as compared to CRC. Conventional endoscopy may be useful in the diagnosis of tumors of the proximal duodenum and very distal ileum. Other endoscopic techniques, such as wireless capsule endoscopy and double balloon endoscopy, can be useful in certain circumstances [24,25]. Wireless capsule endoscopy allows a complete small bowel exploration, but it is contraindicated in the context of an occlusion and does not allow a tissue biopsy, when deemed necessary [24]. Double balloon endoscopy may be useful in patients with small bowel strictures or when a biopsy or a preoperative tattoo is required [25]. Conventional imaging with computed tomography (CT) or magnetic resonance (MR) should be used for staging purpose. However, newer imaging techniques, such as CT or MR enterography or enteroclysis, can be taken into consideration after the failure of conventional imaging, if clinical suspicion persists [26,27].

## 6. Staging

SBA staging is based upon the *AJCC Staging Manual*, *VIII Edition*. T1 tumors invade the lamina propria or the submucosa, T2 tumors invade the muscularis propria, T3 extend into the subserosa or into nonperitonealized perimuscular tissue without serosa penetration, while T4 tumors penetrate the visceral peritoneum or directly infiltrate other organs (e.g., other loops of small intestine or the mesentery of bowel loops). Nodal staging includes N0 (no regional nodal metastases), N1 (one or two positive regional nodes), and N2 (three or more positive nodes). M1 tumors are characterized by distant metastases. Stage I includes T1–2/N0 SBA, while T3–4/N0 define stage II. Stage III disease is defined by the presence of regional lymph nodes metastases, while stage IV SBA has distant metastases. Five-year OS ranges between 57 and 66% in stage I and between 5 and 19% in stage IV [28]. Table 2 summarizes the VIII edition of the TNM staging classification for SBA.

## 7. Management of Locoregional Disease

### 7.1. Surgery

Surgery represents the mainstay of treatment for locoregional disease and the only curative option, with the type of resection depending on tumor location. In general, a segmental resection is the standard surgical procedure, with the exception of duodenal tumors, which often require a pancreaticoduodenectomy, especially if the tumor is located in the second portion of the duodenum or invades the ampulla or the pancreas [29]. Segmental duodenal resection is possible in cases of proximal (first portion of the duodenum) or distal tumors (third portion of the duodenum), non-infiltrating tumors, or tumors of the duodeno-jejunal angle. For tumors located in the jejunum or ileum, segmental resection with lymph node dissection and jejuno-jejunal or ileo-ileal anastomosis is recommended, while for tumors involving the last ileal loop or the ileocecal valve, ileocecal resection or right hemicolectomy with resection of the ileal loop and ligature of the ileocolic artery at its origin allowing an adequate lymph node dissection are the preferred procedures. Generally speaking, the principle of surgical treatment is the resection of the tumor with a distal and proximal margin of at least 5 cm, a negative circumferential margin, and an en-bloc exeresis of the adjoining mesentery with location of the vascular pedicle (distal ganglia), while performing adequate loco-regional lymph nodes dissection [30]. However, the most appropriate surgical approach, in particular for duodenal adenocarcinomas, has been a matter of debate for long time. In a recent analysis from the Surveillance, Epidemiology, and End Results (SEER) database, no difference in terms of overall survival (OS) was observed between radical resection (e.g., in the form of pancreaticoduodenectomy) and simple segmental resection, after controlling for confounding factors on multivariate Cox regression analysis (OR 1.11; 95% CI 0.97–1.27) [31]. Furthermore, segmental resections seem to be associated with a shorter length of hospital stay and less postoperative morbidity [32,33]. Another vexata quaestio concerns the minimum number of lymph nodes that should be retrieved during surgery. Regional lymph nodes are considered to be the retropancreatic, hepatic artery, inferior pancreaticoduodenal, and superior mesenteric for duodenal tumors, and cecal or ileocolic nodes for jejuno-ileal tumors. A number of retrospective series demonstrated that harvesting a higher number of lymph nodes is associated with a longer survival [34,35]. In a SEER analysis, harvesting at least nine lymph nodes for jejunoileal and five lymph nodes for duodenal cancers resulted in the greatest survival difference. Increasing the lymph nodes ratio (LNR) at both sites was associated with decreased median OS (LNR = 0.71 months; LNR 0–0.02, 35 months; LNR 0.21–0.4, 25 months; and LNR > 0.4, 16 months; *p* < 0.001) [36]. Although some data have suggested that a higher number of lymph nodes retrieved may predict better survival [37], international guidelines suggest a minimum of eight locoregional lymph nodes to be assessed for adequate staging [38]. Nevertheless, in published series, 18–44% of patients have six or fewer lymph nodes assessed, with T3–T4 tumors being more frequently associated with nodal metastases [31,39,40].

### 7.2. Adjuvant Treatment

As compared to CRC, SBA (especially if located in the duodenum) has a poorer prognosis, with a 5-year OS ranging from 26% to 41% and with stage III and IV having significantly worse outcomes [29]. In patients with radically resected SBA, the high risk of disease relapse implies the urgent need for effective adjuvant treatments. Roughly 22–42% of patients with resected SBA receive adjuvant chemotherapy, which generally includes a fluoropyrimidine (5-FU or capecitabine) plus or minus oxaliplatin (FOLFOX or XELOX regimen). However, according to National Cancer Database (NCDB), the use of adjuvant chemotherapy has significantly increased over time, rising from 8% of patients in 1985 to 43.4% in 2011 [41,42]. In clinical practice, regimens used for SBA parallel those commonly administered in the adjuvant setting for CRC, although published results, essentially coming from retrospective series or meta-analyses, are conflicting [41,43,44]. Furthermore, data coming from retrospective studies are skewed by the small sample size, the selection bias favoring the administration of adjuvant chemotherapy in higher risk patients and the inadequate chemotherapy regimens used in some studies [45]. Bearing in mind these limitations, adjuvant chemotherapy seems to benefit patients with stage III, T4 disease, or positive margins [39,46]. An international, randomized trial (BALLAD) is currently ongoing, with the aim to clarify the role of adjuvant chemotherapy in patients with radically resected SBA (NCT02502370). Patients with stage I–III SBA are randomly assigned to receive either adjuvant chemotherapy (5-FU plus leucovorin or FOLFOX) or observation. Enrollment in this trial is strongly encouraged with the hope of shedding light on this controversial issue. Surprisingly, also stage I tumors are eligible for enrollment in this trial. In resected SBA, systemic relapse largely predominates above local recurrence. Indeed, in a large retrospective series, local and distant relapse rates were 18% and 86%, respectively [47]. However, owing to its retroperitoneal location, a higher incidence of locoregional failure has been observed in duodenal adenocarcinomas, thus advocating the use of chemo-radiotherapy in this setting [32]. In fact, in an analysis of the US NCDB, 11% of resected SBA received adjuvant radiotherapy, with or without chemotherapy, mainly for duodenal tumors [48]. Furthermore, in a propensity score-matched analysis of the US NCDB of patients with resected, non-metastatic duodenal adenocarcinoma who received adjuvant chemotherapy or chemo-radiotherapy, the latter was more frequently used for patients who underwent positive-margin surgical resection (15.9% vs. 9.1%; *p* < 0.001). However, no survival advantage was observed for patients who were treated with adjuvant chemo-radiotherapy compared with those who received adjuvant chemotherapy (median OS: 48.9 months vs. 43.5 months [HR, 1.04; 95% CI 0.88–1.22 (*p* = 0.669)]), even in high risk cases (positive-margin, T4, inadequate lymph node staging, lymph node positivity or poorly differentiated histology) [49]. Therefore, given the limited data about the efficacy of chemo-radiotherapy in this setting, it should be considered only in selected cases after multidisciplinary discussion. 

In early-stage CRC, MSI identifies a subgroup of tumors with a better prognosis, while in metastatic disease it seems to confer a negative prognosis. Furthermore, a large amount of preclinical and clinical evidences suggest a possible resistance to 5-FU-based chemotherapy in these tumors. The outcome of 5-FU-based chemotherapy in MSI-H CRC is unclear, if not detrimental, especially in stage II [19]. Adjuvant 5-FU-based chemotherapy in resected MSI-H SBA could theoretically be spared, at least in stage II disease. However, since no specific data have been published in this setting, caution should be used when extrapolating data from a different disease.

Furthermore, in an unprecedented international effort, a recent pooled analysis of six phase III randomized trials in patients with stage III CRC treated with a combination of fluoropyrimidine and oxaliplatin demonstrated, in particular for patients with low risk disease (T1–3, N1) and treated with a XELOX regimen, that 3 months of chemotherapy as compared to 6 months offers almost identical benefits, with markedly less toxicity [50]. However, taking in consideration the poorer outcome as compared to CRC and the absence of published data in the specific setting of SBA, the standard duration of adjuvant chemotherapy in SBA remains 6 months.

## 8. Management of Advanced Disease

### 8.1. Systemic Therapy

Owing to non-specific symptoms and difficult radiological detection, approximately one-third of patients diagnosed with SBA have stage IV disease at presentation, with a 5-year relative survival of only 42% [34,51]. In addition, a high proportion of those with resected stage I–III disease ultimately relapses, with the most common sites of metastases being the liver and the peritoneum [47]. 

Although data supporting chemotherapy use in patients with advanced SBA are mainly derived from retrospective series and small phase II studies, it seems generally to be beneficial, with response rates ranging between 6% and 50% and a median OS of 9–19 months [47,52,53,54,55,56]. Overall, the best results seem to be correlated with the use of oxaliplatin-based doublets (FOLFOX or XELOX), which thus became the recommended front-line regimen [57,58,59,60,61]. The clinical value of adding the anti-VEGF monoclonal antibody bevacizumab to first-line chemotherapy has been explored in retrospective series [62,63,64] and in a single-center phase II trial in which 30 patients with SBA and ampullary adenocarcinoma were treated with XELOX regimen plus bevacizumab [65]. The response rate (RR) was 48.3%, with 1 complete response (CR) and 13 partial responses (PR); 10 patients achieved stable disease (SD). At a median follow-up of 25.9 months, the median PFS was 8.7 months (95% CI, 4.9–10.5 months) and the median OS was 12.9 months (95% CI, 9.2–19.7 months). Treatment was well tolerated, and the most common grade 3 toxicities were fatigue (23%), hypertension (23%), neutropenia (20%), and diarrhea (10%). Recently, Amano and colleagues evaluated a series of 74 patients with advanced SBA treated with palliative chemotherapy plus bevacizumab [66]. Immunostaining was performed for vascular endothelial growth factor-A (VEGF-A), TP53, Ki67, β-catenin, CD10, MUC2, MUC5AC, MUC6, and MMR proteins. Patients with high VEGF-A expression and those who received platinum-based chemotherapy with bevacizumab as a first-line treatment had significantly longer PFS and tended to have longer OS than those treated without bevacizumab (*p* = 0.025 and *p* = 0.056, respectively), whereas no differences were observed in patients with low VEGF-A expression. However, the potential predictive role of VEGF-A expression for bevacizumab efficacy in SBA deserves further confirmation in larger series. Furthermore, whilst in CRC triplet with FOLFOXIRI in combination with bevacizumab became one of the standard regimens in the first-line setting, it has not been formally studied in SBA [67]. However, in a phase II trial, 33 patients with advanced SBA were treated with the CAPIRINOX combination, with a modulation of the irinotecan dose according to UDP-glucuronosyltransferase (UGT)1A1*28 genotypes [68]. The regimen yielded a confirmed RR of 37.5% (95% CI, 21%–56%), with a median PFS of 8.9 months and a median OS of 13.4 months. Neither hematologic toxicity (grade ≥ 3 in 52.9%, 30.0%, and 33.3%, respectively, of the 6/6, 6/7, and 7/7 genotype groups) nor tumor RR (41.2%, 33%, and 33%, respectively) were found to differ significantly by UGT1A1 genotype. However, this regimen raises some concerns about hematological and gastrointestinal toxicity and deserves further evaluation in larger studies aiming at comparing its efficacy and toxicity with oxaliplatin-based doublets.

In the second-line setting, published data are even more scarce. In a retrospective study, 28 patients with SBA pre-treated with a platinum-based regimen were treated with a FOLFIRI regimen. The overall response rate (ORR) was 20%, and disease control rate (DCR) was 52%. Median PFS and OS were 3.2 and 10.5 months, respectively. Grade 3–4 toxicity was observed in 48% of patients (grade 3–4 neutropenia, 37%) [69]. Based on these data, FOLFIRI represents a reasonable option for second-line therapy. Furthermore, since genomic data have demonstrated SBA to be a genetically unique entity, even drugs not traditionally utilized in CRC deserve to be explored. In a single-center retrospective study, 20 patients were treated with taxane-based regimens (monotherapy in 3, combination therapy in 17 patients). Median time to progression (TTP) was 3.8 months (95% CI: 2.9–4.6), and median OS was 10.7 months (95% CI: 3.1–18.3) [70]. A phase II study evaluated the efficacy of nab-paclitaxel in the treatment of refractory SBA. Among the 10 assessable patients, 2 achieved a PR, with a median PFS of 3.2 months and a manageable safety profile [71]. Overall, these results demonstrate clinical activity from taxane-based therapy in advanced SBA. Of note, nab-paclitaxel has not been approved in Europe for this indication.

Pembrolizumab is a PD-1 inhibitor whose efficacy was evaluated in a phase II study of patients with pre-treated metastatic solid tumors subdivided in three cohorts: (1) deficient(d)MMR CRC, (2) proficient(p)MMR CRC, and (3) dMMR cancer other than CRC (included 2 SBAs). The immune-related objective RR and immune-related PFS rate were 40% (4 of 10 patients) and 78% (7 of 9 patients), respectively, for dMMR CRC and 0% (0 of 18 patients) and 11% (2 of 18 patients) for pMMR CRC. The median PFS and OS survival were not reached in the cohort with dMMR, but were 2.2 and 5.0 months, respectively, in the cohort with pMMR CRC (HR for disease progression or death, 0.10 [*p* < 0.001], and HR for death, 0.22 [*p* = 0.05]). Patients with dMMR non-CRC had responses similar to those of patients with dMMR CRC (immune-related objective RR, 71% (5 of 7 patients); immune-related PFS rate, 67% (4 of 6 patients)) [72]. Based on these data, FDA granted accelerated approval for the first tissue/site agnostic indication. In the recently published ZEBRA trial, 40 patients with advanced, pre-treated SBA received pembrolizumab 200 mg i.v. every 3 weeks until disease progression, toxicity, or 35 doses maximum. With three confirmed PR (8%; 95% confidence interval (CI), 2–20), this phase II trial did not meet the predefined success criteria of ORR 30%. Median PFS and OS were 2.8 (95% CI, 2.7–4.2) and 7.1 months (95% CI, 5.1–17.1), respectively. However, one confirmed PR (3%) was seen in a patient with MSI-low(L)/MSS tumor and correlated with high tumor mutation burden (TMB). Fifty percent of patients with MSI-H tumors achieved PR and remained alive without progression. Therefore, despite this study not meeting the pre-specified RR, some responses were observed in biomarker-selected patients [73]. Likewise, in a pilot phase II study aiming at evaluating the safety and efficacy of Avelumab in pre-treated SBA, eight patients were enrolled. Of seven efficacy-evaluable patients, two had a PR for an RR of 29%; the DCR was 71% (5/7). Median PFS was 8.0 months. Most frequent, related adverse events were fatigue (38%), elevated alkaline phosphatase (25%), and infusion-related reactions (25%), all ≤ G2; a G3 (not serious) hypokalemia and a G4 (serious) diabetic ketoacidosis occurred in 1 patient each. Despite this benefit, accrual was slower than expected, and the study was closed early, probably because a high number of patients receiving immunotherapy off-label [74].

Moreover, a small phase II trial with panitumumab in patients with SBA was stopped early due to futility [75], confirming once again the unique molecular profile of SBA as compared to CRC or other gastrointestinal malignancies. Table 3 summarizes the results of the most relevant phase II studies in patients with advanced SBA.

### 8.2. Surgery of Primary Tumor and Metastatic Disease

Overall, in asymptomatic patients with metastatic disease, primary tumor resection is not recommended. Palliative surgeries or stenting can be considered after a case-by-case multidisciplinary evaluation, taking into consideration the prognosis, the burden of metastatic spread, and the urgency to start a systemic treatment. 

Data concerning liver metastasectomy in patients with SBA are extremely limited. In a retrospective series of patients who underwent liver metastasectomy for tumors other than CRC and neuroendocrine neoplasia, 28 jejuno-ileal and 12 duodenal adenocarcinomas were included. Five-year survival and OS were 49% and 58 months, respectively, for jejuno-ileal and 21% and 34 months for duodenal adenocarcinomas, respectively [76]. Furthermore, in a recent French multicenter series, 34 patients undergoing resection of metastatic SBA were analyzed. The sites of metastases were peritoneum (29.4%), liver (26.5%), lymph nodes (11.8%), lung (2.9%), multiple (14.7%), and other (14.7%). Thirty (88.2%) patients received adjuvant or perioperative chemotherapy. The median OS was 28.6 months, and relapse-free survival (RFS) was 18.7 months. Fourteen (41.2%) patients survived for more than 36 months [77]. Therefore, in selected patients with limited metastatic disease and favorable prognostic factors, surgery of metastases can be considered, following discussion within an experienced multidisciplinary team.

Peritoneal carcinomatosis (PC) affects approximately one third of SBAs, more frequently those originating from the jejunum and ileum, and carries a poor prognosis, with median OS of 5.9 months [78,79]. Systemic treatment represents the primary option for these patients, although the prognosis remains dismal. An alternative option in patients with isolated PC consists of cytoreductive surgery (CRS) and hyperthermic intraperitoneal chemotherapy (HIPEC), which, over the last decades, has progressively gained in popularity for the treatment of peritoneal surface malignancies, such as peritoneal mesothelioma or pseudomixoma peritonei, and peritoneal metastases from colorectal, gastric, or ovarian cancer [80]. Data about this treatment modality in patients with PC from SBA are essentially derived from retrospective studies, with a reported median DFS of 10–12 months, an OS of 16–47 months, and a grade III–V morbidity of 12–35% [81,82,83,84,85,86,87]. However these studies are flawed by the small number and the selection of the patients enrolled and a wide heterogeneity in terms of performance status, peritoneal cancer index (PCI), completeness of surgery, intra-abdominal temperature, chemotherapy regimens, duration of HIPEC, and administration of adjuvant chemotherapy. Therefore, there is an urgent need of high-quality evidence supporting this treatment modality. However, given the rarity of the disease, it is unlikely that it will result from prospective trials. Multi-institutional registries could presumably represent the best way to gain more insights into the efficacy and morbidity of CRS plus HIPEC in the treatment of PC from SBA.

## 9. Conclusions and Future Perspectives 

**Figure 2:**
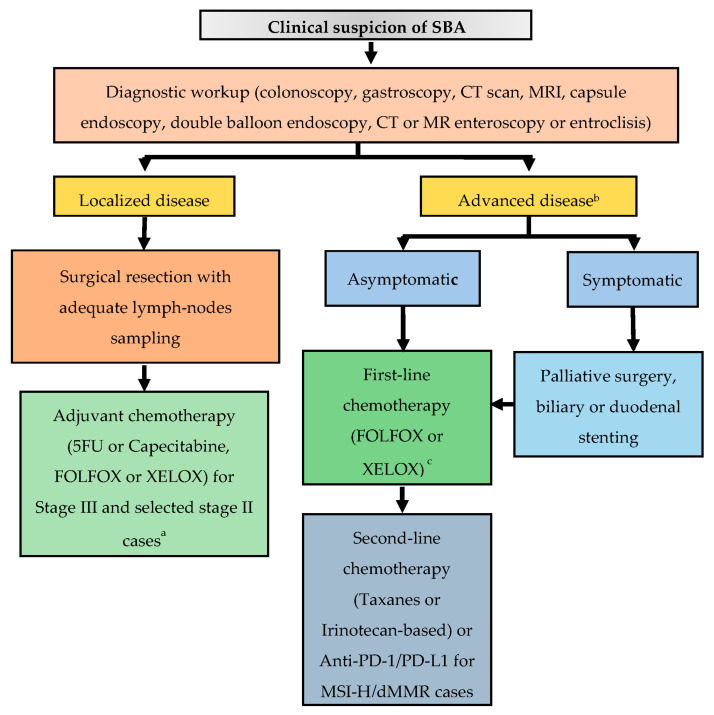
Suggested treatment algorithm in patients with a clinical suspicion of SBA. Abbreviations: CT, computed tomography; MRI, magnetic resonance imaging; FOLFOX, 5-FU, oxaliplatin; XELOX, capecitabine, oxaliplatin; PD-1, programmed death-1; PD-L1, programmed death-ligand 1; MSI-H, microsatellite instability-high; dMMR, deficient mismatch repair. ^a^ Chemo-radiotherapy for selected cases of duodenal tumors, after multidisciplinary discussion. ^b^ Genomic profiling for enrollment in clinical trials with matched target therapies is strongly encouraged. ^c^ Surgery of metastatic sites can be considered in selected cases with oligometastatic disease, after multidisciplinary discussion.

SBA is a rare malignancy, which, owing to its anatomic proximity to the large bowel, has historically been perceived to have a clinical behavior similar to CRC. However, aside its lower incidence and worse prognosis, comprehensive molecular profiling data speak in favor of its uniqueness. Owing to non-specific symptoms and the lack of screening programs, even in high-risk individuals, this disease is often diagnosed at an advanced stage. In patients with localized disease, surgical resection with adequate lymph node sampling represents the only treatment with a chance of cure. However, up to a third of patients with resectable disease experiences a relapse. Therefore, in particular for patients at higher risk of relapse, adjuvant chemotherapy with a fluoropyrimidine-based regimen is recommended, although definitive data on its efficacy are lacking. Enrollment in the BALLAD trial (NCT02502370), which will hopefully clarify the role of adjuvant chemotherapy in resected SBA, is strongly encouraged. Patients with stage IV disease are usually treated with systemic chemotherapy, which includes oxaliplatin-based doublets in the first-line setting and taxanes or irinotecan-based regimens in the second-line setting. Of note, a phase II, open-label, randomized trial is actually ongoing with the aim to compare the FOLFIRI regimen with paclitaxel plus ramucirumab in patients with pre-treated locally advanced or metastatic SBA (NCT04205968). Data about surgical resection of metastatic disease are extremely limited, and cases with potentially resectable oligometastatic disease should be discussed within experienced multidisciplinary teams. Recent in-depth genomic characterization of a large series of SBAs demonstrated its unique molecular profile as compared to neighboring GC and CRC [16]. Of note, potential targetable genomic alterations were identified in 91% of cases (including HER-2 amplifications/mutations, EGFR amplifications/mutations, MEK1 and BRAF mutations, and PI3K pathway activating alterations), thus suggesting further therapeutic options in an orphan disease of approved targeted agents. As genomic profiling techniques become more available in the near future, enrollment in clinical studies with innovative designs, especially basket trials, aiming at assessing the outcome of matched target therapies will be of paramount importance. On the other hand, developing new preclinical models to test potential therapeutic strategies will be equally crucial. Despite the great expectations after the introduction of immune checkpoint inhibitors (ICIs) in the therapeutic armamentarium of various neoplasms, aside for MSI-H tumors, clinical trial results in the specific setting of SBA have been quite disappointing. However, in the work by Schrock and colleagues [16], a substantial proportion of MSS SBAs had a high TMB, a potential biomarkers of ICIs efficacy. Overall, the fraction of TMB-high SBAs was 9.5%, significantly higher than that of GC or CRC, thus suggesting further investigations on the role of ICIs in SBA. Figure 2 provides an algorithm for the management of patients with a clinical suspicion of SBA.

## Figures and Tables

**Table 1:** Comparison of the most frequent gene alterations between SBA, GC, and CRC (adapted from [16]).

Gene	SBA	GC	CRC
*TP53*	58.4%	58.4%	75%
*KRAS*	53.6%	14.2%	52%
*APC*	26.8%	7.8%	75.9%
*SMAD4*	17.4%	5.2%	18.9%
*PIK3CA*	16.1%	17.7%	12.5%
*CDKN2A*	14.5%	14.7%	2.6%

Abbreviations: SBA, small bowel adenocarcinoma; GC, gastric cancer; CRC, colorectal cancer.

**Table 2:** AJCC TNM staging classification for SBA VIII edition.

	T	N	M
Stage I	1–2	0	0
Stage IIA	3	0	0
Stage IIB	4	0	0
Stage IIIA	Any	1	0
Stage IIIB	Any	2	0
Stage IV	Any	Any	1

**Table 3:** Efficacy outcomes of phase II studies in patients with advanced SBA.

Author [Ref.]	*N*	Regimen	Line	Response Rate (%)	PFS (Months)	OS (Months)
Gibson [56]	38	FAM	≥1	18.4	5	8
Overman [61]	30 ^a^	XELOX	1	50	11.3	20.4
Xiang [59]	33	mFOLFOX	1	48.5	7.8	15.2
Horimatsu [60]	24	mFOLFOX	1	45	5.9	17.3
McWilliams [68]	33	XELIRINOX	1	37.5	8.9	13.4
Gulhati [65]	30 ^b^	XELOX + bev	1	48.3	8.7	12.9
Overman [71]	13 ^c^	Nab-Paclitaxel	≥2	20	3.2	10.9
Gulhati [75]	9 ^d^	Panitumumab	>1	0	2.4	5.7
Pedersen [73]	40	Pembrolizumab	>1	8	2.8	7.1
Cardin [74]	8 ^e^	Avelumab	>1	29	8	NA

Abbreviations: N, number of patients; PFS, progression-free survival; OS, overall survival; FAM, 5-FU, doxorubicin, mytomicin C; XELOX, capecitabine, oxaliplatin; mFOLFOX, 5-FU, oxaliplatin; bev, bevacizumab; NA, not available. ^a^ Eighteen patients with SBA and 12 patients with ampullary adenocarcinoma; ^b^ 23 patients with SBA and 7 patients with ampullary adenocarcinoma; ^c^ 10 patients included in the efficacy-assessable population; ^d^ 9 patients with SBA and 1 patient with ampullary adenocarcinoma; ^e^ 5 patients with SBA, and 3 patients with ampullary adenocarcinoma.
